# mTORC1 Signaling in Oocytes Is Dispensable for the Survival of Primordial Follicles and for Female Fertility

**DOI:** 10.1371/journal.pone.0110491

**Published:** 2014-10-22

**Authors:** Nagaraju Gorre, Deepak Adhikari, Rebecca Lindkvist, Mats Brännström, Kui Liu, Yan Shen

**Affiliations:** 1 Department of Chemistry and Molecular Biology, University of Gothenburg, Gothenburg, Sweden; 2 Department of Obstetrics and Gynecology, Sahlgrenska Academy, University of Gothenburg, Gothenburg, Sweden; State Key Laboratory of Reproductive Biology, Institute of Zoology, Chinese Academy of Sciences, China

## Abstract

The molecular mechanisms underlying reproductive aging and menopausal age in female mammals are poorly understood. Mechanistic target of rapamycin complex 1 (mTORC1) is a central controller of cell growth and proliferation. To determine whether mTORC1 signaling in oocytes plays a direct role in physiological follicular development and fertility in female mice, we conditionally deleted the specific and essential mTORC1 component *Rptor (regulatory-associated protein of mTORC1)* from the oocytes of primordial follicles by using transgenic mice expressing *growth differentiation factor 9 (Gdf-9)* promoter-mediated Cre recombinase. We provide *in vivo* evidence that deletion of *Rptor* in the oocytes of both primordial and further-developed follicles leads to the loss of mTORC1 signaling in oocytes as indicated by loss of phosphorylation of S6K1 and 4e-bp1 at T389 and S65, respectively. However, the follicular development and fertility of mice lacking *Rptor* in oocytes were not affected. Mechanistically, the loss of mTORC1 signaling in *Rptor*-deleted mouse oocytes led to the elevation of phosphatidylinositol 3-kinase (PI3K) signaling that maintained normal follicular development and fertility. Therefore, this study shows that loss of mTORC1 signaling in oocytes triggers a compensatory activation of the PI3K signaling cascade that maintains normal ovarian follicular development and fertility.

## Introduction

In mammals, the pool of primordial follicles serves as the source of developing follicles and fertilizable ova for the entire reproductive lifespan of the organism [1]. At any given time, only a limited number of primordial follicles are recruited into the growing follicle pool through follicular activation and the majority of primordial follicles remain in a dormant state. Menopause, also known as ovarian senescence, occurs when the pool of primordial follicles has been virtually exhausted [2]–[4]. The duration of fertility and the timing of menopause are thus determined by the size and persistence of the primordial follicle pool [1]–[4].

Mechanistic target of rapamycin (mTOR) is a highly conserved serine/threonine kinase and a member of the PI3K-related kinase family [5]. mTOR nucleates two large physically and functionally distinct signaling complexes: mTOR complex 1 (mTORC1) and mTOR complex 2 (mTORC2) [6]. mTORC1 controls many cellular processes that ultimately determine cell growth, including protein synthesis, ribosome biogenesis, nutrient transport, and autophagy [5], [7]. In mammals, mTORC1 consists of mTOR, Raptor, PRAS40 (proline-rich AKT substrate 40 kDa), mLST8 (mammalian lethal with sec-13 protein 8; also known as GβL), and Deptor (DEP domain-containing mTOR-interacting protein) and is sensitive to rapamycin [7], [8]. It has been shown that Raptor is involved in mediating mTORC1 assembly, recruiting substrates, and regulating mTORC1 activity and subcellular localization. The strength of the interaction between mTOR and Raptor can be modified by nutrients and other signals that regulate the mTORC1 pathway [9]–[12].

Conventional knockout of the *Rptor* gene in mice – which codes for the specific and essential mTORC1 component Raptor – is embryonic lethal [13]. To directly explore the role of mTORC1 in ovarian follicular development and fertility *in vivo*, we generated mice lacking *Rptor* specifically in the oocytes of both primordial and further-developed follicles by using transgenic mice expressing *growth differentiation factor 9 (Gdf-9)* promoter-mediated Cre recombinase. We found that deletion of *Rptor* specifically in the oocytes leads to loss of mTORC1 signaling. However, follicular development and fertility in mice lacking *Rptor* in their oocytes were not affected by the loss of mTORC1 signaling. Interestingly, PI3K signaling was found to be elevated upon the loss of mTORC1 signaling in *Rptor*-deleted oocytes, and this activity is presumed to maintain the follicular development and fertility in these mice.

## Results

### Generation and validation of mutant mice with oocyte-specific deletion of *Rptor*

**Figure 1:**
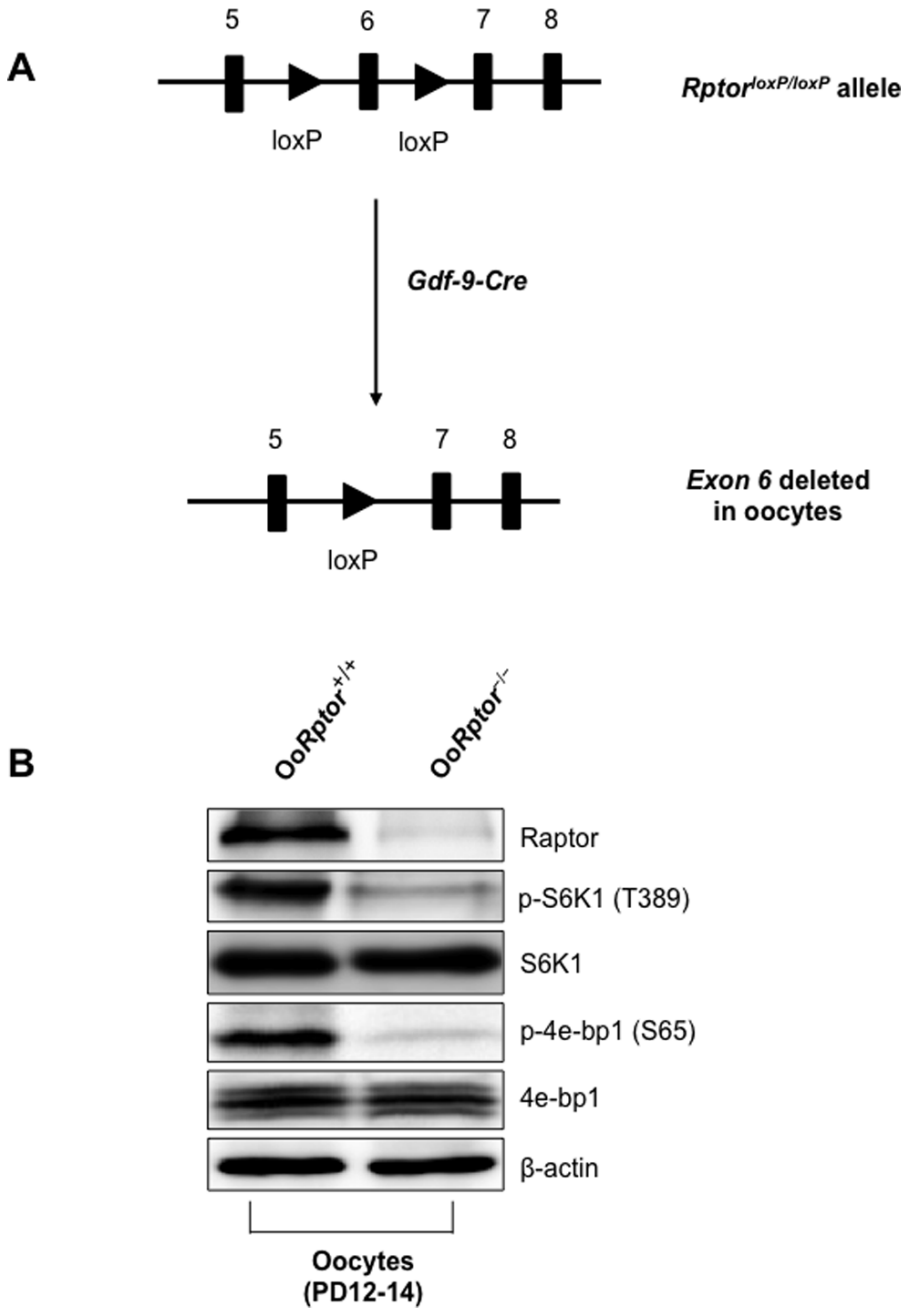
Generation and validation of oocyte-specific deletion of *Rptor* (Oo*Rptor* ^−/−^) in mice. (A) Schematic representation of deletion of *exon 6* of the *Rptor* gene by *Gdf-9-Cre*-mediated recombination in oocytes. (B) Western blots showing the absence of protein expression of Raptor in the oocytes of Oo*Rptor* ^−/−^ mice and the loss of mTORC1 activity as indicated by the loss of phosphorylation of S6K1 and 4e-bp1 at T389 and S65, respectively. Oocytes were isolated from ovaries of PD12–14 Oo*Rptor* ^+/+^ and Oo*Rptor* ^−/−^ mice as described in the Materials and Methods. For each lane, about 20 µg of protein was loaded. Levels of β-actin were used as internal controls. The experiments were repeated three times, and representative images are shown.

To study how mTORC1 in oocytes regulates the activation and development of primordial follicles, we generated mutant mice in which the *Rptor* gene was deleted specifically in the oocytes of primordial and further-developed follicles (referred to as Oo*Rptor* ^−/−^ mice). This was achieved by crossing *Rptor^loxP/loxP^* mice [14] with transgenic mice carrying *Gdf-9* promoter-mediated Cre recombinase [15], [16] (Fig. 1A). To determine the efficiency of deletion of *Rptor* in oocytes, we performed western blot analysis on oocytes collected from postnatal day (PD)12–14 Oo*Rptor* ^−/−^ and Oo*Rptor* ^+/+^ mice. We found that expression of Raptor protein was completely abolished in growing Oo*Rptor* ^−/−^ oocytes (Fig. 1B) indicating successful deletion of the *Rptor* gene from the oocytes. To further validate that the loss of *Rptor* in oocytes leads to loss of mTORC1 signaling in Oo*Rptor* ^−/−^ oocytes, we examined the phosphorylation of its well-known substrates S6K1 and 4e-bp1 [12], [17]. As shown in Fig. 1B, phosphorylation of S6K1 and 4e-bp1 at T389 and S65, respectively, was effectively abolished in the Oo*Rptor* ^−/−^ oocytes indicating that mTORC1 signaling is suppressed in the mutant oocytes.

### Loss of mTORC1 signaling in oocytes does not affect the fertility of female mice

**Figure 2:**
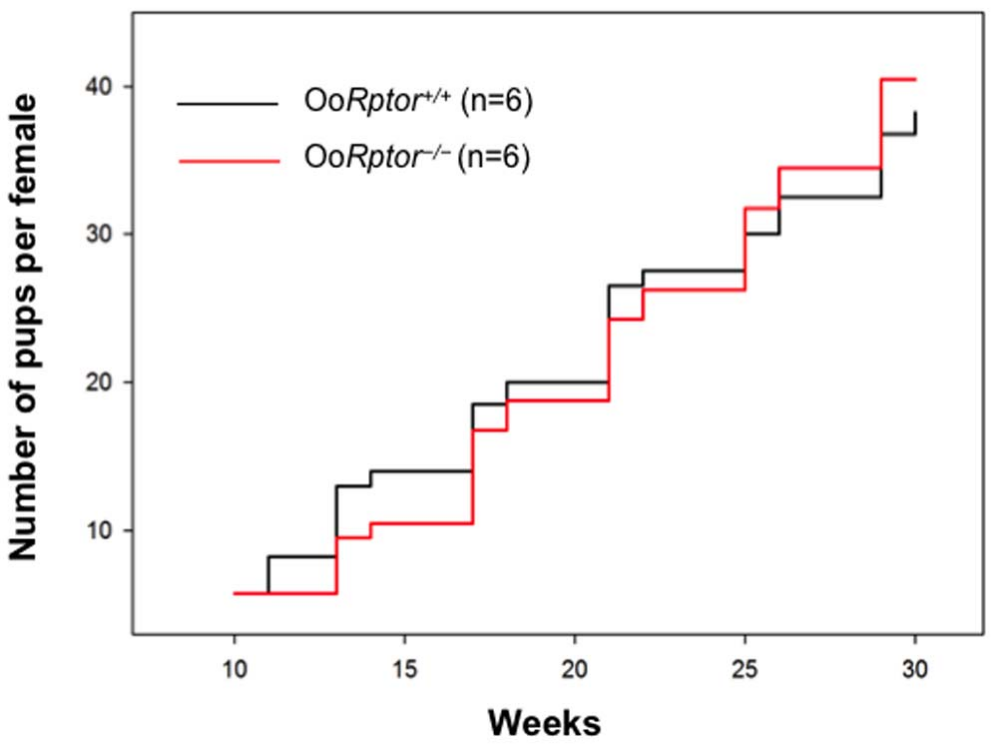
Fertility is not altered in Oo*Rptor* ^−/−^ females. Fertility curve comparing the average cumulative number of pups per Oo*Rptor* ^−/−^ (red line) and Oo*Rptor* ^+/+^ (black line) female. All Oo*Rptor* ^−/−^ females are fertile indicating that loss of mTORC1 from oocytes does not affect the fertility of female mice.

We found that the Oo*Rptor* ^−/−^ females sexually matured and had a normal vaginal opening at the age of 5–6 weeks. To determine whether the loss of mTORC1 signaling from oocytes influences the fertility of Oo*Rptor* ^−/−^ mice, we housed Oo*Rptor* ^−/−^ and Oo*Rptor* ^+/+^ mice with wild-type males. We found that the fertility of Oo*Rptor* ^−/−^ females was comparable to that of Oo*Rptor* ^+/+^ females during the testing period from 6 weeks to 30 weeks of age (Fig. 2). These results show that loss of mTORC1 signaling in oocytes does not affect the fertility of female mice.

### PI3K–Akt signaling is enhanced in Oo*Rptor* ^−/−^ oocytes

**Figure 3:**
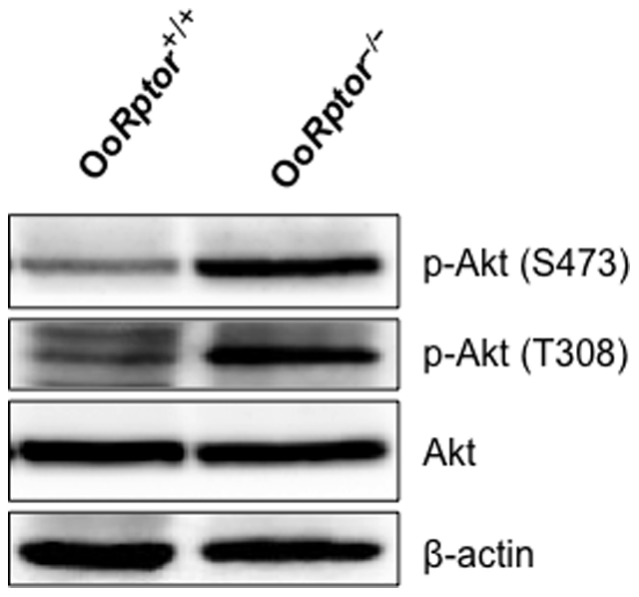
PI3K–Akt signaling in Oo*Rptor* ^−/−^ and Oo*Rptor* ^+/+^ oocytes. Oocytes were isolated from the ovaries of Oo*Rptor* ^−/−^ and Oo*Rptor* ^+/+^ mice at PD12–14, and western blots were performed as described in the Materials and Methods. Levels of phosphorylation of Akt at S473 and T308 are elevated in Oo*Rptor* ^−/−^ oocytes compared to Oo*Rptor* ^+/+^ oocytes, and this indicates that PI3K–Akt signaling in Oo*Rptor* ^−/−^ oocytes is enhanced. Levels of total Akt and β-actin were used as internal controls.

In recent years, the PI3K–Akt signaling cascade in oocytes has been shown to have important roles in controlling the activation and development of ovarian follicles and fertility [16], [18]–[20]. To explore the molecular mechanisms underlying the normal fertility of Oo*Rptor* ^−/−^ mice, we investigated PI3K signaling in Oo*Rptor* ^−/−^ oocytes. We found that the activity of Akt is enhanced in Oo*Rptor* ^−/−^ oocytes as indicated by the hyperphosphorylation of Akt at S473 and T308 (Fig. 3). This demonstrated that the loss of mTORC1 signaling leads to the hyperactivation of PI3K–Akt signaling in Oo*Rptor* ^−/−^ oocytes.

### Elevated PI3K–Akt signaling leads to normal follicular development in Oo*Rptor* ^−/−^ mouse ovaries

**Figure 4:**
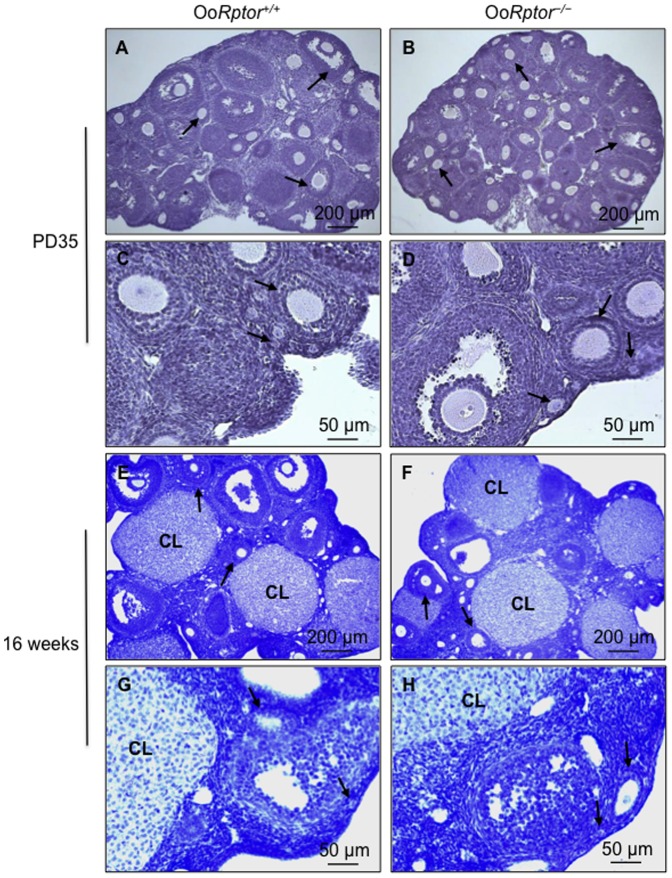
Normal follicular development in Oo*Rptor* ^−/−^ mice. Morphological analysis of ovaries from Oo*Rptor* ^−/−^ and Oo*Rptor* ^+/+^ littermates at PD35 and at 16 weeks of age. Ovaries from Oo*Rptor* ^−/−^ and Oo*Rptor* ^+/+^ mice were embedded in paraffin, and sections 8 µm thick were prepared and stained with hematoxylin. The overall development of follicles (arrows) and corpora lutea (CL) in Oo*Rptor* ^−/−^ mice was found to be normal (B, D, F, and H) compared to Oo*Rptor* ^+/+^ mice (A, C, E, and G).

To investigate whether ovarian follicular development in Oo*Rptor* ^−/−^ mice is normal due to the elevated PI3K–Akt signaling, we studied the morphology of ovaries collected from Oo*Rptor* ^−/−^ and Oo*Rptor* ^+/+^ mice at PD35 and at 16 weeks of age. At PD35, follicles at various developmental stages ranging from primordial to preovulatory were found in Oo*Rptor* ^−/−^ ovaries (Fig. 4B and D), and this was comparable to Oo*Rptor* ^+/+^ ovaries (Fig. 4A and C). In addition, we found healthy corpora lutea along with all types of follicles in Oo*Rptor* ^−/−^ ovaries at 16 weeks of age (Fig. 4F and H), and this was also comparable to Oo*Rptor* ^+/+^ ovaries (Fig. 4E and G). These results show that the loss of mTORC1 signaling in Oo*Rptor* ^−/−^ oocytes leads to elevated PI3K–Akt signaling and that this is sufficient for normal follicle development.

## Discussion

In this study, we used a mouse model with an oocyte-specific deletion of *Rptor* to show that Raptor in oocytes is essential for maintaining mTORC1 signaling in oocytes. Follicular development and fertility in mice lacking *Rptor* in their oocytes were not affected by the loss of mTORC1 signaling, but PI3K signaling was found to be elevated upon the loss of mTORC1 signaling in *Rptor*-deleted oocytes. Due to the elevated PI3K–Akt signaling, ovarian follicular development and fertility were found to be normal in mice lacking *Rptor* in the oocytes of both primordial and further-developed follicles. Therefore, we conclude that loss of mTORC1 signaling in oocytes triggers a compensatory activation of the PI3K–Akt signaling cascade that maintains normal ovarian follicular development and fertility.

In our earlier study, we showed that constitutively enhanced oocyte PI3K–Akt signaling by loss of *Pten (Phosphatase and tensin homolog)* in primordial oocytes, which is the upstream negative regulator of PI3K–Akt signaling, causes global activation of all primordial follicles and premature ovarian failure (POF) [16]. In contrast, oocyte-specific deletion of *Pdk1 (3-phosphoinositide-dependent protein kinase-1)*, which plays a major role in phosphorylating and activating Akt and S6K1 [21], leads to the premature loss of primordial follicles and POF by suppressing Akt–S6K1 signaling. Interestingly, concurrent loss of Pdk1 and Pten in oocytes reverses the global activation of the primordial follicle pool caused by loss of Pten [19]. However, the global activation of primordial follicles in oocyte-specific *Pten* mutant mice (Oo*Pten* ^−/−^) is not completely prevented by treatment with rapamycin *in vivo* [22], which is a well-known pharmacological inhibitor of mTORC1 [8]. Similarly, phosphorylation of Akt is not altered when wild-type oocytes are treated with rapamycin *in vitro* [19]. However, our *in vivo* results demonstrate that loss of mTORC1 signaling in oocytes triggers a compensatory activation of the PI3K–Akt signaling cascade and that this is required to maintain normal ovarian follicular development and fertility.

Deletion of *Tsc1 (tuberous sclerosis complex 1* or *hamartin)* in oocytes (Oo*Tsc1* ^−/−^), which is a negative regulator of mTORC1, also leads to premature activation of the entire pool of primordial follicles and subsequent POF due to the enhanced mTORC1 signaling in oocytes. Such over-activation of primordial follicles is rescued when Oo*Tsc1* ^−/−^ mutant mice are treated with rapamycin *in vivo* [23], [24]. Together with the current paper, our studies indicate that the mTORC1 signaling may not be indispensable for physiological activation of primordial follicles.

In this study, compensatory activation of the PI3K–Akt signaling cascade was observed when Raptor was missing from the oocytes, and this activity appears to be essential to maintain normal physiological follicular development and fertility in Oo*Rptor* ^−/−^ females. Such compensatory activation of PI3K–Akt signaling has been seen in mice with both adipocyte-specific and skeletal muscle-specific ablation of *Rptor* [25], [26]. Our results demonstrate that activation of PI3K–Akt signaling in the absence of mTORC1 signaling in oocytes is required to compensate for this loss and to support physiological development of ovarian follicles and female fertility. Although we observed the elevation of PI3K signaling in the absence of mTORC1 signaling, it is possible that other unidentified factors might contribute to the compensation of the Raptor deletion. Our results suggest the dual inhibition of both mTORC1 and PI3K pathways, which is commonly used to treat certain types of malignancies, might have adverse effect on follicular survival and female fertility.

## Materials and Methods

### Mice

*Rptor^loxP/loxP^* mice [14] in a C57BL/6J genomic background were crossed with transgenic mice carrying *Gdf-9* promoter-mediated Cre recombinase [15], [16] that also had a C57BL/6J background. After multiple rounds of crossing, we obtained homozygous mutant female mice lacking *Rptor* in their oocytes (Oo*Rptor* ^−/−^ mice). Control mice that do not carry the Cre transgene are referred to as Oo*Rptor* ^+/+^ mice. The mice were housed under controlled environmental conditions with free access to water and food. Illumination was on between 0600 and 1800. All animal experiments were approved by the Committee on the Ethics of Animal Experiments of the University of Gothenburg and were carried out in accordance with the approved guidelines.

### Reagents, antibodies, and immunological detection methods

Rabbit monoclonal antibody (EP539Y) to Raptor was purchased from Abcam. Rabbit polyclonal antibodies to phospho-S6K1 (T389), phospho-4E-BP1 (S65), and phospho-Akt (S473) as well as rabbit monoclonal antibodies to S6K1 and 4e-bp1 were obtained from Cell Signaling Technologies (Beverly, MA, USA). Mouse monoclonal antibody to phospho-Akt (T308) was purchased from BD Bioscience (Franklin Lakes, NJ, USA). Mouse monoclonal antibodies to β-actin and paraformaldehyde were purchased from Sigma-Aldrich Sweden AB (Stockholm, Sweden). Western blots were carried out according to the instructions of the suppliers of the different antibodies and visualized using the ECL Prime western blotting detection system (Amersham Biosciences, Uppsala, Sweden). Paraffin and hematoxylin were purchased from Histolab, Sweden.

### Histological analysis

Ovaries were fixed in 4% paraformaldehyde, dehydrated, and embedded in paraffin. The paraffin-embedded ovaries were serially sectioned at 8-µm thickness and rehydrated followed by staining with hematoxylin for morphological observation. Ovarian follicles at different developmental stages were categorized based on the well-accepted standards established by Pedersen and Peters. Ovarian morphology was determined based on images taken with a light microscope (Zeiss Axio Scope A1 upright microscope). One or both ovaries from more than three mice of each genotype were used for each time point.

### Isolation of oocytes from postnatal mice ovaries

Mice were sacrificed by decapitation, and the ovaries were dissected free of fat and connective tissue using a dissection microscope. The ovaries were then minced with a pair of dissection scissors before being incubated in 0.05% collagenase in Dulbecco's modified Eagle's medium-F12 (DMEM/F12; Invitrogen) supplemented with 4 mg/mL bovine serum albumin (BSA), 100 units/mL penicillin, and 100 µg/mL streptomycin. The solution was mixed with frequent agitation and pipetting. After the tissues had mostly been digested by the collagenase, usually within 45–60 min, EDTA was added to this mixture to a final concentration of 40 mM and the mixture was incubated at 37°C with frequent pipetting for another 15–20 min until clusters of granulosa cells or other cells were completely dispersed. The mixture of cells and oocytes was then washed once and cultured in a 6 cm or 10 cm tissue culture dish with the above-mentioned serum-free DMEM/F12 medium for 12 h to allow the granulosa cells and other ovarian cells to attach to the plastic. The unattached oocytes and red blood cells were then recovered by collection of the supernatant and centrifugation at 1300 rpm for 5 min at room temperature. Red blood cells were subsequently removed using a hypotonic buffer containing 144 mM NH_4_Cl and 17 mM Tris–HCl (pH 7.2). After several washes, oocytes were collected by centrifugation. They were then lysed in a buffer containing 50 mM Tris–HCl (pH 8.0), 120 mM NaCl, 20 mM NaF, 20 mM β-glycerophosphate, 1 mM EDTA, 6 mM EGTA (pH 8.0), 1% NP-40, 1 mM DTT, 5 mM benzamidine, 1 mM PMSF, 250 µM sodium orthovanadate, 10 µg/mL aprotinin, 10 µg/mL leupeptin, and 1 µg/mL pepstatin followed by centrifugation at 14,000 rpm for 20 min at 4°C. The supernatants were collected and protein concentrations were measured using the bicinchoninic acid (BCA) protein assay, and equal amounts of proteins were used for western blot.
